# Pleural mesothelioma (PMe): The evolving molecular knowledge of a rare and aggressive cancer

**DOI:** 10.1002/1878-0261.13591

**Published:** 2024-03-08

**Authors:** Manuel Rigon, Luciano Mutti, Michelangelo Campanella

**Affiliations:** ^1^ Centre for Clinical Pharmacology and Precision Medicine William Harvey Research Institute Queen Mary University of London UK; ^2^ Department of Biology University of Rome Tor Vergata Rome Italy; ^3^ Department of Biotechnological and Applied Clinical Sciences DISCAB, L'Aquila University L'Aquila Italy; ^4^ Temple University Sbarro Institute for Cancer Research and Molecular Medicine Philadelphia PA USA; ^5^ Department of Biomedical Sciences University of Padua Padua Italy; ^6^ Institute Gustave Roussy Villejuif France

**Keywords:** asbestos, BAP1 and therapy, mesothelioma

## Abstract

Mesothelioma is a type of late‐onset cancer that develops in cells covering the outer surface of organs. Although it can affect the peritoneum, heart, or testicles, it mainly targets the lining of the lungs, making pleural mesothelioma (PMe) the most common and widely studied mesothelioma type. PMe is caused by exposure to fibres of asbestos, which when inhaled leads to inflammation and scarring of the pleura. Despite the ban on asbestos by most Western countries, the incidence of PMe is on the rise, also facilitated by a lack of specific symptomatology and diagnostic methods. Therapeutic options are also limited to mainly palliative care, making this disease untreatable. Here we present an overview of biological aspects underlying PMe by listing genetic and molecular mechanisms behind its onset, aggressive nature, and fast‐paced progression. To this end, we report on the role of deubiquitinase BRCA1‐associated protein‐1 (BAP1), a tumour suppressor gene with a widely acknowledged role in the corrupted signalling and metabolism of PMe. This review aims to enhance our understanding of this devastating malignancy and propel efforts for its investigation.

## Graphical abstract

Pleural mesothelioma (PMe) is a rare but lethal pathology in which the carcinogen asbestos has a proven causative role. Asbestos travels inside the lungs and airways to prime degeneration of mesothelioma cells. Pathogenic molecular mechanisms and pathways are here outlined as well as ways for clinical intervention for this malignancy, which remains untreatable.

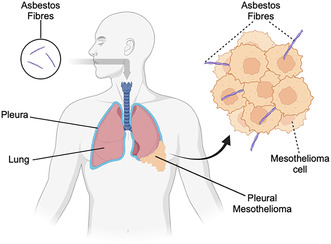

**Abbreviations**
5‐hmC: 5‐hydroxymethylcytosine
AE1/3: pan‐cytokeratin antibody cocktail
AKT: protein kinase B
AP‐1: activator protein 1
ASX: additional sex combs
ASXL1: additional sex combs like 1
ASXL2: additional sex combs like 2
BAP1: BRCA1 associated protein 1
BARD1: BRCA1 associated RING domain 1
BCL‐2: B‐cell lymphoma 2
BRCA1: breast cancer associated protein 1
Ca^2+^
: calcium ions concentration
CDK4: cyclin‐dependent kinase 4
CDK6: cyclin‐dependent kinase 6
CDKN2A: cyclin‐dependent kinase inhibitor 2A
CR: calretinin
CSC: cancer stem cell
DNA: deoxyribonucleic acid
Dvl: dishevelled
EGFR: epidermal growth factor receptor
EMT: epithelial to mesenchymal transition
EPP: extrapleural pneumonectomy
ERK: extracellular signal‐regulated kinases
FOXK1: forkhead box protein K1
FOXK2: forkhead box protein K2
GSH: glutathione
HCF1: host cell factor C1
HEG1: heart development protein with EGF like domains 1
ICIs: immune check points inhibitors
IHC: immunohistochemistry
IMRT: intensity modulated radiotherapy
IP3R2: type 3 inositol‐1,4,5‐trisphosphate receptor
IP3R3: type 3 inositol‐1,4,5‐trisphosphate receptor
MBD5: methyl‐CpG binding domain protein 5
MBD6: methyl‐CpG binding domain protein 6
MCL‐1: induced myeloid leukaemia cell differentiation protein 1
Merlin: moesin‐ezrin‐radixin‐like protein
miRNA: micro RNA
MIS: mesothelioma *in situ*
MMe: malignant mesothelioma
mTOR: mammalian target of rapamycin
NF2: neurofibromin 2
OCT4: octamer‐binding transcription factor 4
OVT: oncolytic viral therapy
P/D: pleurectomy/decortication
p14^ARF^
: alternative open reading frame of CDKN2A gene in *Homo sapiens*
p16^INK4A^
: cyclin‐dependent kinase inhibitor p16
p19^ARF^
: alternative open reading frame of CDKN2A gene in *Mus musculus*
p53: tumour protein p53
pAKT: phosphorylated protein kinase B
PDT: photodynamic treatment
PMe: pleural mesothelioma
PRC1: polycomb repressor complex 1
PR‐DUB: polycomb repressive deubiquitinase
PTEN: phosphatase and tension homologue
RNA: ribonucleic acid
ROS: reactive oxygen species
RTK: receptor tyrosine kinase
SLC7A11: solute carrier family 7 member 11
SOMAscan: slow Off‐Rate modified Aptamer proteomic analysis
TNF‐α: tumour necrosis factor alpha
TPDS: tumour predisposition syndrome
UCH: ubiquitin carboxy‐terminal hydrolase
ULD: UCH37‐like domain
WNT: wingless and INT‐1
WT1: Wilms' tumour 1
YAP1: yes‐associated protein 1
YY1: Yin Yang 1

## 1 Introduction

**Fig. 1:**
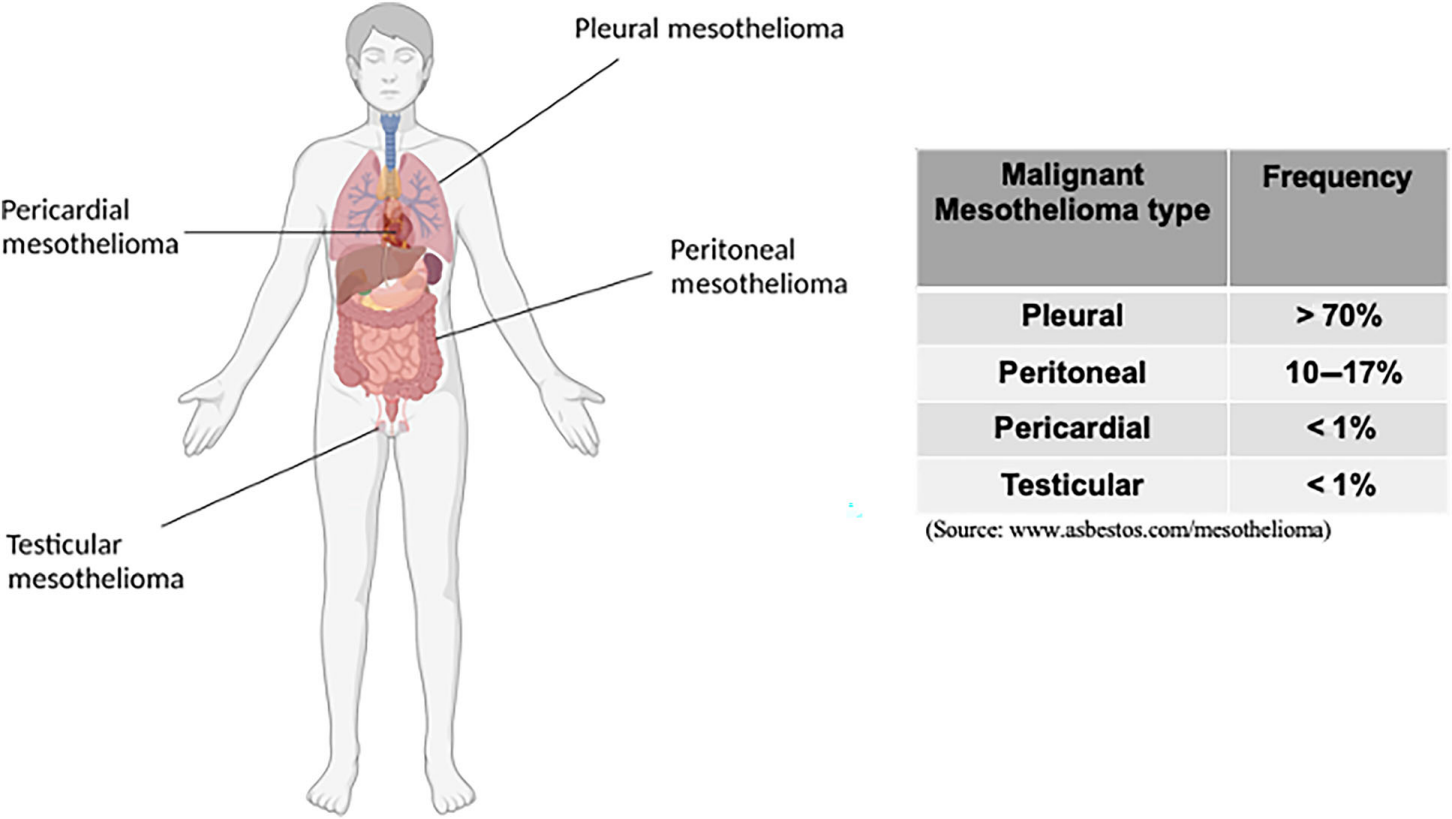
Tissues affected by mesothelioma. Representation of the most common tissues of origin for the mesothelioma pathology. Source: www.asbestos.com/mesothelioma. Created with Biorender.com.

**Fig. 2:**
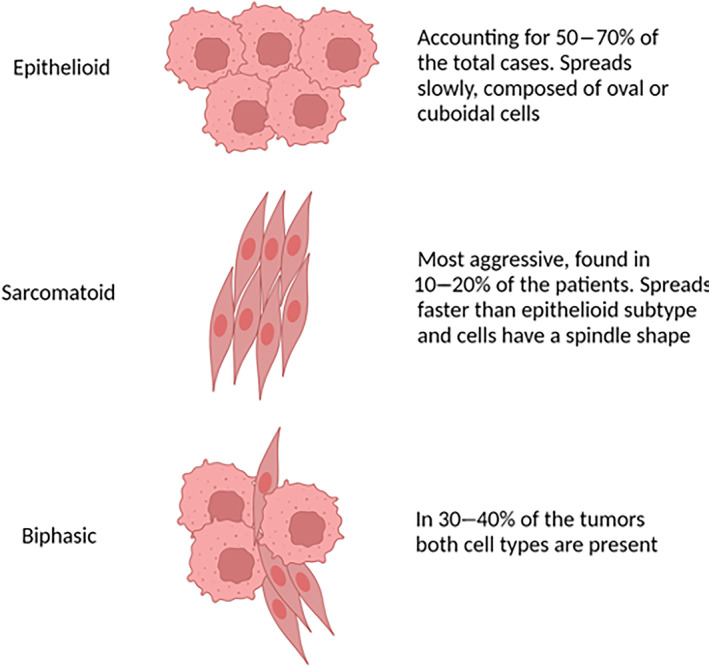
Mesothelioma cell types. Illustration of the three possible cell types involved in mesothelioma. Created with Biorender.com.

The mesothelium refers to the layer of tissues (epithelium) that surrounds most body cavities and the organs of the chest (pleura and pericardium), abdominal cavity (peritoneum and mesentery), and pelvis (including the tunica vaginalis that surrounds the testes) [1]. It functions to protect internal structures and aid in movement and breathing. Such functions are supported by the cells—called mesothelial cells—which are commonly found in pleural, peritoneal, and pericardial fluids [2]. Mesothelial cells tend to have a large, round, and centrally placed nucleus with a generous amount of basophilic cytoplasm. In response to inflammation or infection, mesothelial cells lose their cytoplasm and converge into clusters. Several medical problems may involve the mesothelium and its resident population such as pleural and pericardial effusions, adhesions, and malignant mesothelioma (MMe) [3]. MMe has an incidence rate ranging between 7 and 40 cases per million [4], killing approximately 40 000 people worldwide per year [5]. Currently, there is a lack of suitable treatments to revert its fatal prognosis as well as of reliable markers allowing for an early diagnosis. MMe develops in lining tissues of the body such as the pericardium, tunica vaginalis, peritoneum, and the pleura [6] (Fig. 1). Pleural mesothelioma (PMe), which includes the parietal layer of the internal chest cavity and the visceral line which covers the lungs [7], accounts for more than 70% of all MMe cases. PMe can develop on either layer of the pleura and spreads to the other [8], resulting in the growth of the tumour around the affected lung, which can lead to breathing difficulties caused by the accumulation of pleural fluids in the thoracic cavity [9]. Importantly, PMe is histologically distinguishable according to the cell type involved in the pathology [10], i.e. (a) epithelioid, (b) sarcomatoid, or (c) biphasic [11] (Fig. 2). Epithelioid mesothelioma is the most common type, tends to be less aggressive, and spreads more slowly compared to sarcomatoid and biphasic ones [12]. Sarcomatoid mesothelioma is associated with the worst prognosis and is the most aggressive and difficult to treat of the three types, accounting for 10–20% of the PMe cases [13]. The biphasic type is a mix of the previous two [14] and its prognosis may depend upon which cell type is most abundant in the tumour. Epithelioid PMe is composed of polygonal, oval, or cuboidal cells, whilst sarcomatoid PMe cells have a spindle shape [15]. The most widely accepted method currently to define PMe pathology progression is the Tumour Node Metastasis (TNM) staging system [16]. See Box 1 on Tumour Node Metastasis (TNM) classification.

Box 1 **TNM Classification uses three components to identify cancer stage**

- *Tumour extent (T)*: The location and size of the primary tumour site, which can range from T0 if there is no evidence of the tumour, to T4 when the tumour has spread from the pleura to nearby tissues and organs.
- *Lymph node involvement (N)*: Whether the cancer has spread to nearby lymph nodes, from N0 to N2 based on how far from the primary tumour the affected lymph nodes are.
- *Metastasis (M)*: M0 for no evidence and M1 the for presence of distant metastases, depending on whether the tumour is localised or has spread to distant areas of the body.

The clinician will then score each component and assigns a value. A combination of component scores establishes the mesothelioma stage, between I and IV.

- *Stage IA*: T1, N0, M0
- *Stage IB*: T2 or 3, N0, M0
- *Stage II*: T1 or 2, N1, M0
- *Stage IIIA*: T3, N1, M0
- *Stage IIIB*: T1‐3, N2, M0 *or* T4, any N, M0
- *Stage IV*: any T, any N, M1

Assessment of cancer progression through the staging system is essential to plan the best possible treatments, but as of today a cure for this pathology has yet to be achieved. For this reason, the average life expectancy for PMe patients is between 8 and 22 months after diagnosis [17], depending on stage and histological subtype. Symptoms include dry cough, shortness of breath, and chest pain [4], which often appear at a late stage of the pathology and since these are common symptoms of other diseases, they may lead to misdiagnosis [18].

This occurs despite an awareness of the main risk factor of the pathology is exposure to asbestos [6]. Even though genetic background [19] and simian virus (SV40) [20, 21] infections have been contemplated as triggers of the condition, accumulation of asbestos in respiratory routes is the prominent one. Asbestos is a term used to describe different mineral species [22], of which the most common is chrysotile. Chrysotile, also known as white asbestos [23], makes up 99% of the total types of asbestos produced worldwide [24]. White asbestos has been widely used in the past century in the textile and building industries, due to its durability and physical properties [22, 25], its usage has been nonetheless banned in more than 65 countries since 1970 for the threat it poses to human health via its fibre release into the air [26, 27]. Asbestos modifies DNA and induces chronic inflammation [28, 29], thereby impairing lung function. However, the damage caused to an organism can take up to 50 years to manifest and PMe is indeed a cancer characterised by a very long latency period [30]. The incidence peak of the pathology is believed to have happened already [31] in the Western countries that have banned the use of the mineral. However, highly populated countries such as China, Russia, and India [32] are continuing the mining of asbestos and its usage in construction indicates that the number of PMe patients is expected to rise globally.

Given the growing global attention to this disease, we review what is known about PMe by detailing (a) mechanisms of carcinogenesis, (b) signalling pathways, (c) proven genetic determinants besides diagnostics, and (d) treatments. This overview represents a core‐based knowledge of the disease, which is aimed at assisting both researchers and clinicians.

## 2 Asbestos‐induced PMe pathogenesis

**Fig. 3:**
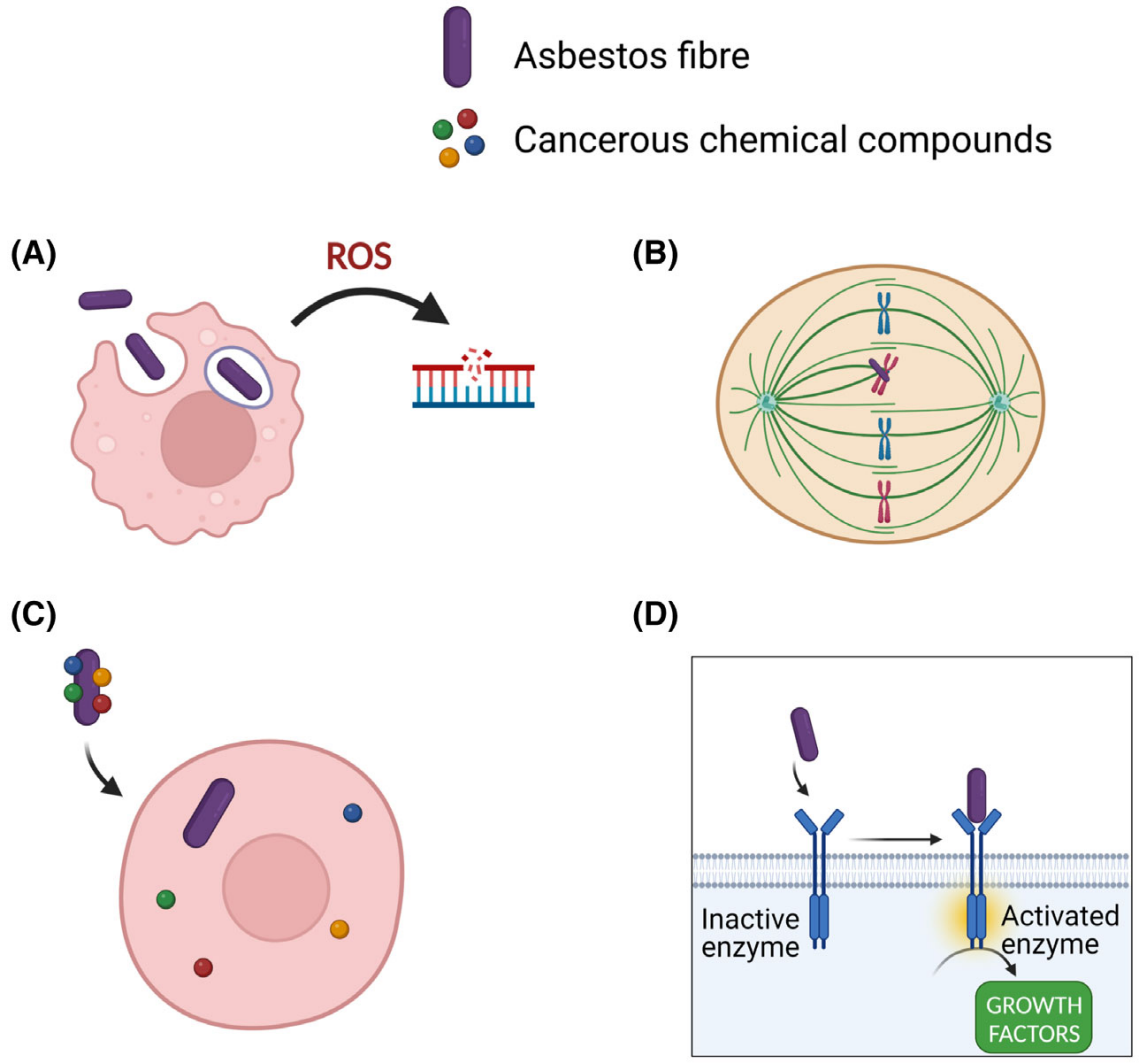
Possible mechanisms of carcinogenesis caused by asbestos. Representation of the proposed mechanisms of action of asbestos fibres in mesothelioma progression. (A) DNA damage caused by ROS produced by macrophages after asbestos phagocytosis. (B) Abnormal cell cycle due to asbestos fibres entangling chromosomes during mitosis. (C) Activation of receptors localised on the cell surface by asbestos fibres. (D) Transport of cancerous chemical compounds inside the cell carried on the surface of the fibres. Created with Biorender.com.

The mesothelium is an important structure that serves not only to protect and lubricate movements of organs, but also facilitates fluid transport, blood clotting, and helps resistance against infections and cancers. While it can aid in controlling tumours, it is also particularly sensitive to asbestos [3]. The first line of evidence showing that asbestos exerts regulatory effects in pleural mesothelial cells came from a study in which asbestos fibres caused the induction of *c*‐*fos* and *c‐jun* proto‐oncogenes [33] in human cells. It is yet ill‐defined how asbestos influences the biology of mesothelial cells, but the currently accepted view is that the induced cytotoxicity leads to DNA damage and/or frustrates phagocytosis, triggering chronic inflammation [34, 35]. During the latency period, from exposure to the carcinogen and pathology outbreak, several core aspects of mesothelial cells change, spanning chromosomal integrity and epigenetic modifications (e.g. promoter hypermethylation at tumour suppressor loci) [36, 37]. Currently, there are four main hypotheses regarding the pathogenesis of asbestos‐induced mesothelioma (Fig. 3A–D).

- The first one pertains to the production of a large amount of reactive oxygen species (ROS). ROS are dangerous oxygen forms, and are highly reactive due to their unstable nature [38]. These molecules can be produced directly from the exposed surface of the fibre, or by phagocytic cells such as macrophages, which engulf asbestos fibres but are not able to digest them (Fig. 3A). ROS can then interact with DNA, leading to DNA damage lesions and mutations. It is nonetheless important to consider that it might be difficult for ROS to reach the DNA in their active state, as they would probably react with membranes and molecules on their way to the nucleus. Therefore, one of the other three mechanisms below, or a combination of them, could better explain the specific action of these dangerous fibres on the cells.
- The second hypothesis indicates that asbestos fibres are engulfed directly by mesothelial cells and can physically interfere with the mitotic process of the cell cycle (Fig. 3D). Tangling of asbestos fibres with chromosomes or mitotic spindles may result in chromosomal structural abnormalities during cell division [39] (Fig. 3B).
- The third one proposes that asbestos traps other molecules on its surface [40], such as proteins and other chemical compounds, resulting in the accumulation of hazardous molecules, including carcinogens (Fig. 3C).
- Finally, the fourth hypothesis suggests that mesothelial cells and macrophages exposed to asbestos fibres release a variety of cytokines and growth factors, such as tumour necrosis factor‐α, interleukin‐1β, transforming growth factor‐β, and platelet‐derived growth factor, which induce inflammation and tumour promotion [41]. This is probably due to the ability of asbestos to directly interact and activate surface receptors (Fig. 3D). Indeed, it has been demonstrated that asbestos fibres activate the epidermal growth factor receptor (EGFR) in mesothelial cells and in turn the downstream extracellular signal‐regulated kinases (ERK) [42] cascade. Moreover, the activation of the pathway is coupled by an upregulation of EGFR mRNA and protein levels [43], leading to an amplification of the signal. One of the principal effects following the upregulation of the ERK cascade in PMe is the activation of the transcription factor activator protein 1 (AP‐1) [44], which controls several cellular processes, such as cellular proliferation, differentiation, and apoptosis [45].

Therefore, asbestos exhibits pleiotropic effects, linked to aberrant transcriptional responses, cell proliferation, and transformation. Either through direct interactions or via the generation of ROS, asbestos activates cell signalling pathways that regulate gene expression and cell fate.

## 3 Pathogenesis beyond asbestos

Even though approximately 80% of individuals developing PMe have been previously exposed to asbestos, fewer than 5% of asbestos workers develop PMe [46]. This suggests that genetic traits may be relevant for the development of the pathology. For instance, a higher incidence of PMe has been observed in certain families among residents exposed to asbestos [47]. Cytogenetic studies have shown that mesothelioma cells carry highly complex and variable chromosomal aberration. However, other pathogenic mechanisms have been unveiled and are reported below.

### 3.1 Chromosomal and genetic changes

Loss‐of‐heterozygosity analyses have demonstrated frequent deletions of specific sites within chromosome arms 1p, 3p, 6q, 9p, 13q, 15q, and 22q [48]. Three of these regions are most frequently altered: the tumour suppressors cyclin‐dependent kinase inhibitor 2A (CDKN2A) at 9p21, neurofibromin 2 (NF2) at 22q12, and the BRCA1‐associated protein 1 (BAP1) at 3p21.1. Homozygous deletion appears to be the major mechanism affecting CDKN2A, whereas inactivating mutations coupled with allelic loss occur at the NF2 locus [48]. More recently, BAP1 was determined to be the gene involved in the mutations or deletions at 3p21.1 [49]. BAP1 is a tumour suppressor protein [50], mainly by acting on DNA repair, cell death, and gene transcription. The *CDKN2A* locus on chromosome 9p21 encodes two tumour suppressor proteins, namely p16^INK4A^ and p14^ARF^ [51]. Mesothelioma, as opposed to many other cancers, rarely has mutated *TP53* [52]. However, loss of p14^ARF^ indirectly inactivates p53, since the degradation of p53 is blocked by p14^ARF^ activity [53]. P53 is an important tumour suppressor, essential for the regulation of cell division, senescence, and apoptosis [54]. The protein p16^INK4A^, instead, controls cell cycle and division by suppressing CDK4 and CDK6 activity [55]. Moreover, in murine models it has been shown that hypermethylation of *p16* ^*INK4A*^ or *p19* ^*ARF*^ (orthologous of the human *p14* ^*ARF*^) precedes mesothelioma accompanied by silencing of CDKN2A and loss of p16^INK4A^ and p19^ARF^ proteins, suggesting that epigenetic alterations may play an important role in gene regulation leading to PMe [37].

### 3.2 Merlin signalling

Moesin‐ezrin‐radixin‐like protein (Merlin) is the protein encoded by the *NF2* gene that affects multiple signalling cascades, including cell adhesion, small GTPases, receptor tyrosine kinase (RTK), Hippo, and the mammalian target of rapamycin (mTOR) pathways [56]. Given its plethora of functions, the loss of Merlin in PMe has been linked not only to increased proliferation, but also increased invasiveness, spreading, and migration [56]. In patients with no detectable *NF2* mutations, Merlin was found to be phosphorylated at Ser518, and thus inactivated [57], confirming the role of Merlin in PMe development.

More specifically, its role in PMe seems to be tightly linked to Hippo and mTOR signalling cascades. Besides the recurring mutations of the LST1 and LST2 kinases, arising from loss of *NF2* gene function [56], Merlin interplays with the Hippo pathway by influencing the transcriptional co‐activator Yes‐associated protein 1 (YAP1). The latter is known to be involved in mesothelial cell growth through the upregulation of cell cycle‐promoting genes [58]. YAP1 activity is normally inhibited by Merlin, through phosphorylation and cytoplasmic retention [59].

### 3.3 Noncoding miRNAs

Not only conventional coordinators of gene transcription such as transcription tactors have been linked to PMe onset, but also microRNAs (miRNAs). miRNAs are small, single‐stranded, noncoding RNA molecules containing 21–23 nucleotides that regulate gene expression by blocking the translation of target messenger RNA (mRNA) [60]. It has been demonstrated that several miRNAs are differentially expressed in mesothelioma cells compared to normal or immortalised mesothelial cells [61, 62]. Most of the differences involve the downregulation of miRNA directed at silencing proto‐oncogenes such as BCL‐2, OCT4, MCL‐1, and others [63]. This leads to a deregulation of essential cellular processes, such as differentiation, proliferation, apoptosis, and metabolism [64]; hence, promoting cancer progression. The downregulation of miRNA expression in PMe is usually due to chromosomal aberrations [63] (e.g. miR‐31 [65]). However, not all differences in miRNA expression are related to a downregulation, given that some miRNAs are abundantly overexpressed in PMe cells [61], such as those targeting CDKN2A and NF2 [61]. Deregulation of the miRNA processing could therefore hold pathogenic relevance in PMe patients.

### 3.4 mTOR and AKT signalling

There are several signalling pathways exploited by PMe during onset and rapid progression. The mammalian target of rapamycin (mTOR) is an important serine/threonine protein kinase, involved in fundamental processes such as autophagy, mitochondrial biogenesis, protein and lipid biosynthesis, and growth. Its defective function in PMe is linked with the fast progression of the disease as well as with its late onset [66]. Recently, it was demonstrated how pharmacologically targeting the mTOR complex [67] it is possible to inhibit malignant cell growth *in vitro* and *in vivo* tumour tissues. mTOR‐regulating signals are predominately linked to a lack of nutrients (specifically amino acids) [68] and are negatively regulated by Merlin, which is repressed by phosphorylated AKT (pAKT) [69, 70]. The constant activation of AKT in PMe could be due to the absence of phosphatase and tension homologue (PTEN), which is either mutated or completely absent in some cases [71, 72]. PTEN acts as a tumour suppressor by negatively regulating intracellular levels of phosphatidylinositol‐3,4,5‐trisphosphate, and phosphorylation levels of AKT [73]. Notably, the undetectable level of this protein in PMe might not only arise from mutations but also from hyperactivation of the Notch‐1 signalling pathway, which controls the transcriptional regulation of PTEN [74]. Moreover, a further mechanism of inactivation of this protein could be related to the effect of asbestos fibres on ROS production since PTEN is also frequently inactivated by H_2_O_2_ [75].

### 3.5 Calretinin and C‐met signalling

The sustained activation of AKT leading to Merlin repression and mTOR exploitation in PMe has been linked to calretinin (CR) [76, 77], which is overexpressed in certain types of tumours and pathologies, including colon carcinoma and PMe [78]. Mostly characterised as an intracellular Ca^2+^ effector and buffer [79], its expression promotes cellular growth, survival, and invasiveness inducing the epithelial to mesenchymal transition (EMT) [73]. In murine cortical neurons, it has been demonstrated that CR expression is regulated by an AP2‐like element present in the promoter region of the gene [80]. This element does not influence CR transcription in PMe [81], indicating different regulation between neuronal and non‐neuronal cells. A further activator of the AKT/mTOR pathway in PMe is speculated to be the hepatocyte growth factor receptor (c‐Met), the dysregulation of which contributes to the regulation of cell growth, motility, and invasion, as well as confers tumours the ability to metastasize [82]. Expressed in most PMe patients but not in healthy mesothelial cells [83], its inhibition by a specific drug (PHA‐665752) resulted in the arrest of the cell cycle and reduction of the activity of AKT and ERK signalling pathways [84].

### 3.6 NF‐kB and WNT signalling

In PMe, the proteasome is also overexpressed, which in combination with the activation of AKT causes activation of the nuclear translocator factor k‐light chain enhancer of activated B‐cells (NF‐kB) [44, 85]. In human mesothelial cells exposed to asbestos fibres [86], NF‐kB is translocated to the nucleus, resulting in the expression of prosurvival genes such as BCL‐2 [87]. A further stimulus for NF‐kB activation in PMe is tumour necrosis factor‐α (TNF‐α), which is abundant in PMe [86]. The Wingless and INT‐1 (WNT) pathway is also deregulated in PMe. The WNT signalling pathway regulates developmental processes, cell proliferation and polarity, and its upregulation is caused by overexpression of the activating protein Dishevelled (Dvl) [88]. Among the proteins capable of redesigning cancer cell signalling, greater consideration for its prominence in PMe was gained by the BRCA1‐associated protein 1 (BAP1). BAP1 is one of the most mutated genes in PMe. Loss of BAP1 nuclear staining is considered a reliable indicator of malignancy, particularly for epithelioid histology, and therefore part of the panel for the differential diagnosis.

**Fig. 4:**
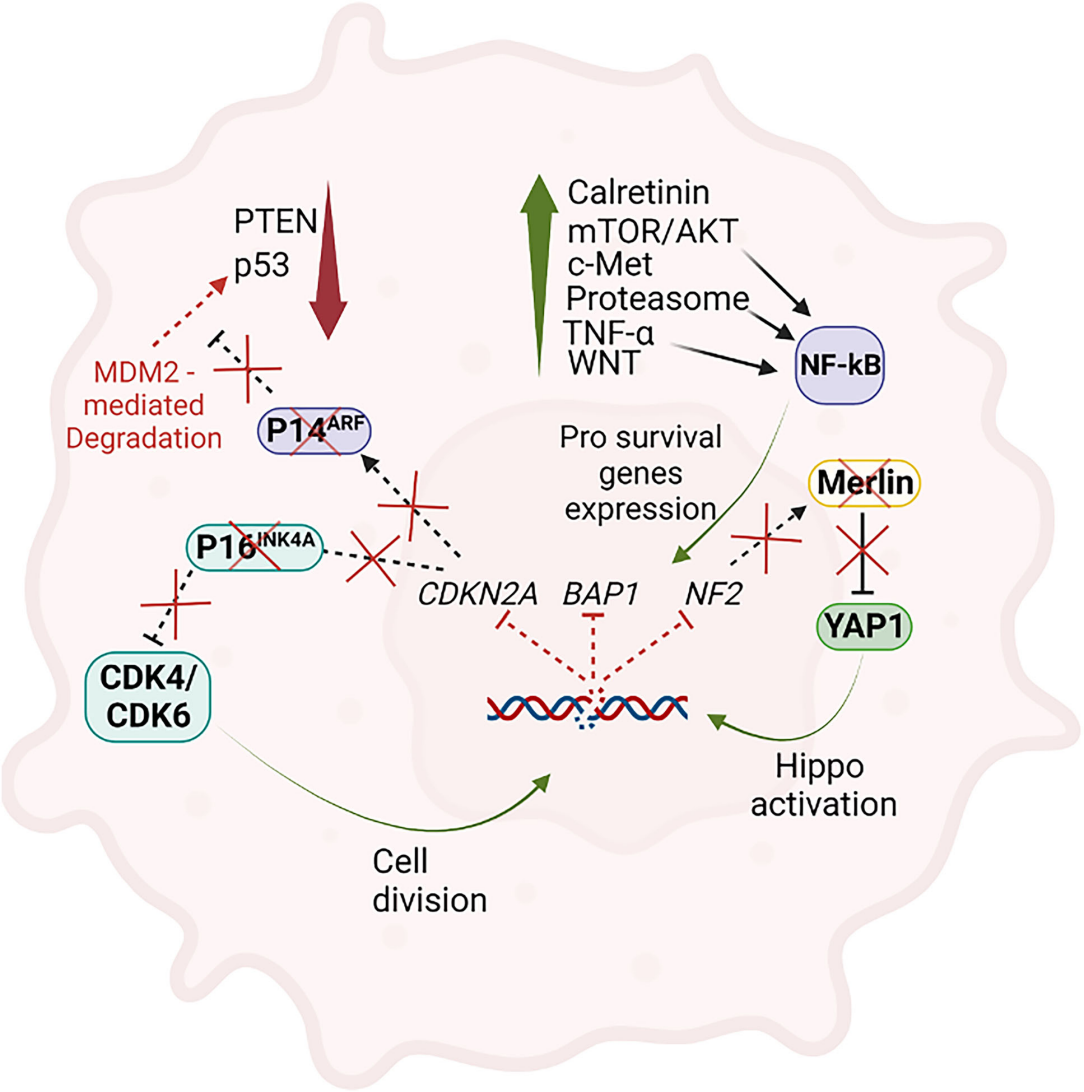
Schematic representation of the genes and protein most commonly deregulated in PMe. Representation of a PMe cell with dysregulated pathways and molecular mediator differently expressed or regulated during the onset of the pathology indicated. Created with Biorender.com.

All this highlights that PMe pathogenesis is multifactorial, mirrored by the complex genetic which characterises the transformed cells causing the disease (Fig. 4).

## 4 Role of the BRCA1‐associated protein 1 (BAP1) in malignant mesothelioma

**Fig. 5:**
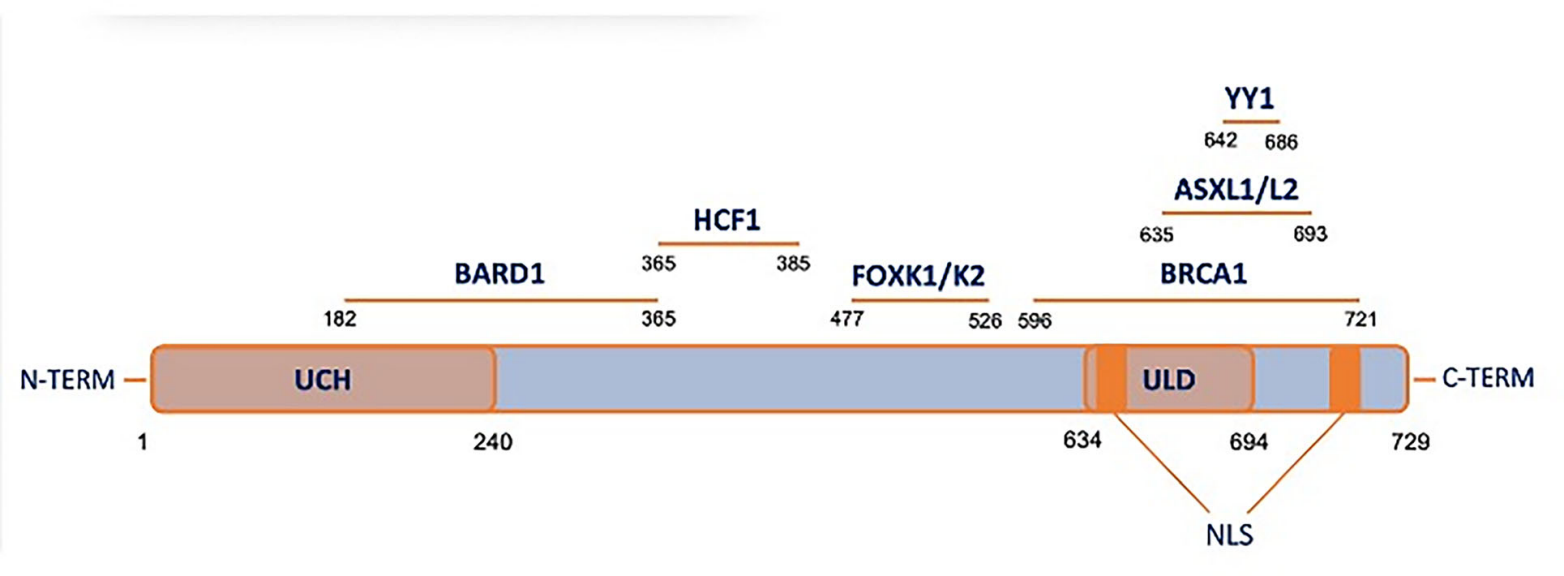
Schematic representation of BAP1 sequence, domains, and binding sites. Representation of BAP1 illustrating the functional domains and known binding sites to other proteins and complexes. UCH, ubiquitin carboxyl‐terminal hydrolase domain; ULD, UCH37‐like domain; NLS, nuclear localization sequence.

BAP1 is a ubiquitin carboxy‐terminal hydrolase of 729 aa, encoded in humans by the BAP1 gene [89]. In its native form it assumes a molecular mass of 80.4 kDa and its structure contains three domains: (a) a catalytic carboxy‐terminal hydrolase (UCH) domain, localised at the first 240 aa of the N‐terminus, which removes ubiquitin from ubiquitylated substrates; (b) a linker region, which includes a host cell factor C1 (HCF1) binding motif; and (c) the C‐terminal region, which comprises a UCH37‐like domain (ULD) and two nuclear localization sequences [90, 91] (Fig. 5). BAP1 was discovered in 1998, following a two‐hybrid screening and named after its interaction with breast cancer‐associated protein 1 (BRCA1) [92]. BRCA1, by interacting with BRCA1‐associated RING domain 1 (BARD1) forms a tumour suppressor complex that regulates the DNA damage response via its E3 ubiquitin ligase activity [93].

**Fig. 6:**
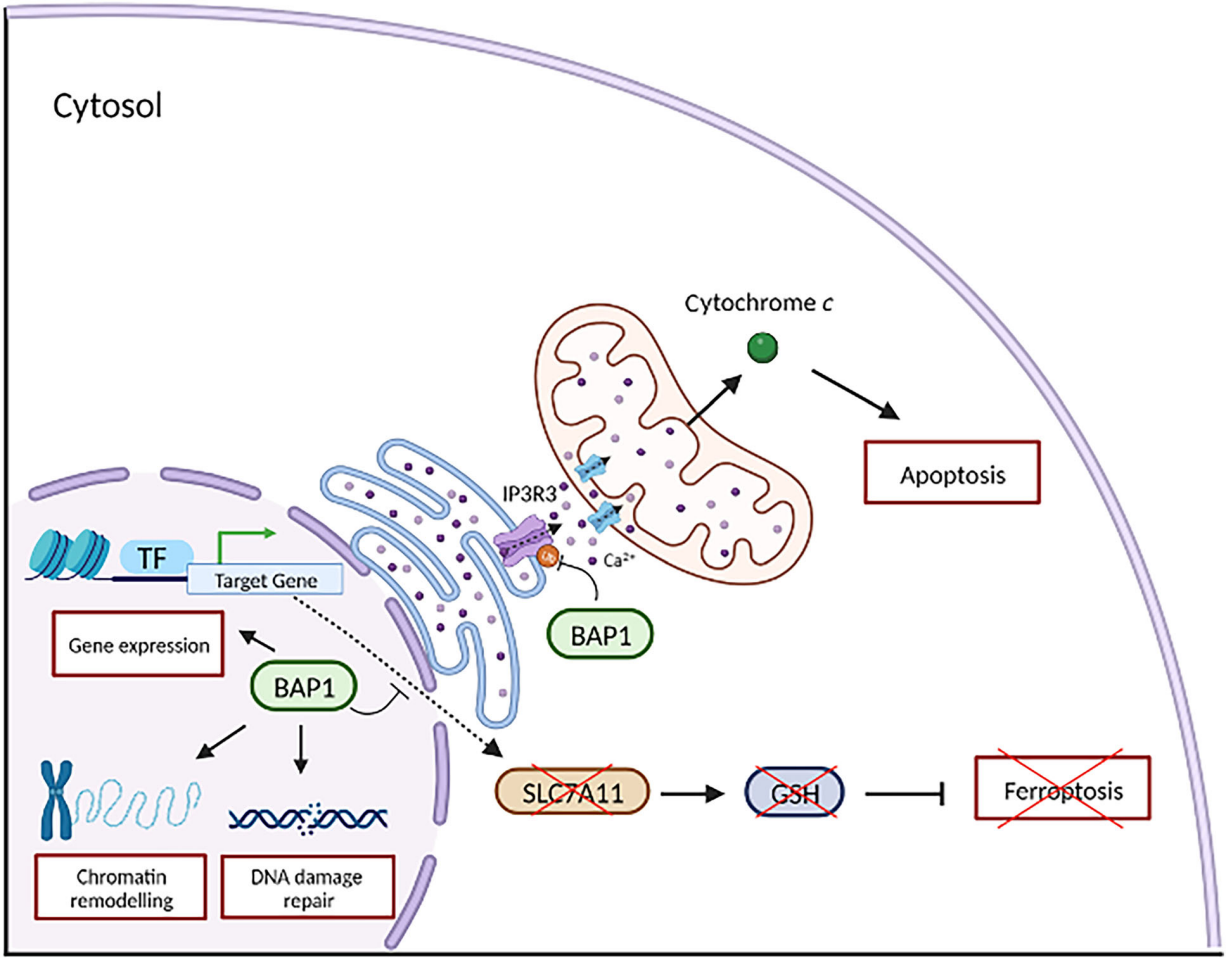
Scheme of the different BAP1 functions in the cell. BAP1 is a tumour‐suppressor protein that exerts its role via the coordination of several different cellular mechanisms. In the nucleus, it is involved in gene expression regulation, DNA damage repair, and chromatin remodelling, while in the cytosol it can stabilise the calcium channel IP3R3. The downstream effects on a cell include the regulation of cell death via apoptosis and ferroptosis. Created with Biorender.com.

BAP1 associates with the complex by binding and deubiquitylating BARD1 [94], thereby modulating the DNA damage response of BRCA1–BARD1. BAP1 interacts with several other proteins in the cell [95, 96, 97] and its DNA repair activity could also be linked to its interaction with the proteins MBD5 and MBD6 [98]. Contrary to what was originally thought, BAP1 localization and activity are not exclusively nuclear, and can also be found in the cytosol [99] (Fig. 6). Given its protective role, BAP1 loss is established as “a foe” when it comes to tumour susceptibility and development. However, patients carrying germline BAP1 mutations in PMe [100, 101, 102] and cutaneous melanoma [103] show an improved survival and a better prognosis, suggesting that the presence of a mutated BAP1 might mitigate tumour aggressiveness (a “friendly” aspect). Moreover, in uveal melanoma, somatic BAP1 mutations cause metastasis with a probability of 74%, while the same has been observed in only 36% of the patients carrying a germline mutation [104]. A possible explanation for the differences reported is that in patients carrying a germline mutation, often a somatic mutation is also observed, thus probably inducing a complete inactivation of the protein [105]. What is clear is that PMe in carriers of *BAP1* mutations are almost exclusively of the epithelioid subtype [101, 106], they are well‐differentiated, and have an overall nonaggressive morphology, consistent with prolonged survival. In addition, even though wildtype BAP1 affects sensitivity to gemcitabine [107], very recent work has shown that mutation of the gene as well as its ablation improves the response to platin/pemetrexed [108].

The question of how the lack of a tumour suppressor protein like BAP1 can lead to an improvement in patient's outcome remains open. This positive effect may be due to the involvement of Ca^2+^ homeostasis in tumour progression. It has been reported that a few Ca^2+^ signalling effectors promote the cancer stem cell (CSC) state and are associated with cell resistance to cancer treatments [109]. In liver cancer, for example, Ca^2+^ oscillation mediated by IP3R2 plays a central role in CSC self‐renewal [110]. The recent findings on the role of BAP1 in the stabilisation of the IP3R3 receptor may support this theory. It is therefore possible indeed that malfunctions in BAP1, even though leading to cancer progression, may prevent the formation of CSCs, thereby reducing the aggressiveness of the cancerous cells.

## 5 Targets for molecular diagnosis

### 5.1 Diagnostic protein markers

Histological analysis is usually not sufficient for PMe diagnosis. It may indeed be hard to distinguish sarcomatoid mesothelioma from sarcomatoid carcinoma of the lung [111] and epithelioid mesothelioma from adenocarcinoma or reactive mesothelial cells [15]. Therefore, an immunohistochemistry (IHC) approach is required using antibodies specific for PMe biomarkers. Even though biomarkers with 100% sensitivity and specificity [112] for MMe are still missing, a few promising candidates have been discovered. Namely, by using a combination of Wilms' tumour 1 (WT1) protein, CAM5.2, and AE1/3 cytokeratins, it is possible to distinguish between sarcomatoid carcinoma and sarcomatoid mesothelioma with a specificity of 97.7% [113]. CR is also a useful diagnostic protein to distinguish mesothelioma from adenocarcinoma, with a sensitivity of 95% and specificity of 87% [114]. Another promising readout is the heart development protein with EGF‐like domains 1 (HEG1), which for the epithelioid subtype offers a sensibility comparable to CR, but with a higher specificity [115]. The discrimination between benign mesothelial proliferation and epithelial mesothelioma is rather difficult. To this end, the evaluation of the nuclear absence of BRCA1‐associated‐protein 1 (BAP1) and 5‐hydroxymethylcytosine (5‐hmC) emerges as a valid strategy [116]. Furthermore, a loss of BAP1 has been associated with mesothelioma *in situ* (MIS) [117]: a condition currently acknowledged as a precursor to PMe with an epithelioid component [118].

It is worth mentioning that there are blood‐based biomarkers that can help inform diagnosis, thus aiding the development of a screening methodology beyond classical histology, perhaps allowing for an early detection of PMe. To this goal, fibulin‐3 has been proposed as a possible detectable protein in the serum, even though there is still controversy that would explain a lack of attention towards this blood‐based marker [119]. Mesothelin is present in the blood too, and a study conducted on more than 4000 [120] patients has shown that it could be used as a serum marker to confirm PMe due to its high specificity (95%), although a sensitivity score of 32% does not advocate for this protein as a suitable diagnostic tool. However, encouraging recent studies have demonstrated that when mesothelin is scored in combination with CR, it is possible to reach a sensitivity of 75% [121] without losing any specificity.

### 5.2 The role of microRNAs as diagnostic markers

The screening for sarcomatoid mesothelioma via a blood test remains challenging, since it appears to be quite difficult to find specific protein markers linked to a histological subtype. Hope in this direction is brought by the detection of miRNAs. Previous analysis of tissue samples showed that for the correct diagnosis of PMe, the detection of differentially expressed miRNA [62] stands as a valid option. The deregulation of several miRNAs is a characteristic of PMe, regardless of histologic subtypes [122], and an assay validated on 68 samples based on the detection of three miRNAs reached a sensitivity of 100% and a specificity of 94% against adenocarcinoma [123]. In addition, it has been shown that miRNA and DNA molecules can be released from the cells into the body fluids [124, 125], in which they are remarkably stable, being protected from endogenous RNAse activity [126]. In keeping with this, the circulating upregulated microRNAs miR‐197‐3p, miR‐1281, and miR‐32‐3p have been proposed as potential new biomarkers [127] in PMe, but further studies will be needed to prove the efficacy of such a screening methodology for the detection of both epithelioid and sarcomatoid cell types. Lastly, for the same purpose, a novel interesting screening technique termed SOMAscan proteomics has been proposed, in which the presence of thousands of proteins in the serum is scored simultaneously. Recently, it has been shown that it is possible to detect PMe with a 75% sensitivity and 88.2% specificity using this method [128], which is an encouraging result but still not enough by itself. Perhaps soon, it will be possible to increase the sensitivity and specificity of this assay by producing a better screening array for this pathology.

## 6 Therapeutic tools

There is no cure for PMe and most of the current strategies of intervention are palliative, aimed at extending the lifespan of the patients. The multimodality treatment that sees surgery followed by radiotherapy and chemotherapy is the adopted standard of care, even though high incidences of failure and recurrence are still registered [129].

A recent cross‐sectional study has found that the incidence of PMe's death toll is constantly increasing worldwide, and particularly so in regions with limited resources [130]. Furthermore, the survival rate 5 years postdiagnosis is around 10% [131], corroborating that currently available treatments for PMe have little impact. Patient candidates recommended for radical surgery represent a lower percentage (around 20%) [132] and those are younger individuals. The surgical procedures consist of: (a) extrapleural pneumonectomy (EPP), *en‐bloc* resection of the lung, pleura, pericardium, and diaphragm, or (b) the pleurectomy/decortication (P/D), a lung‐sparing surgery that involves removing the tumours and the affected pleura [133]. A cure is not generally achieved following EPP [134], so the liberation of the tumour mass with P/D in a multimodal treatment scenario has been mostly adopted in recent years [135], mainly as a palliative [136].

The utility of P/D is still under debate and a recent study in the UK was conducted to assess whether the adoption of this procedure is effectively advantageous in terms of survivability and quality of life [137], but the results have yet to be published. It is important to note that although the multimodality treatment including surgery, chemotherapy and radiotherapy has been proposed for many years, it is not indicated as a standard of care [136].

Radiotherapy alone does not appear to be effective in resolving the pathology for PMe [138], although intensity‐modulated radiotherapy (IMRT) following surgery [139, 140] is mainly beneficial in easing the pain and symptoms.

Chemotherapy too shows marginal efficacy in PMe [136], considering the high resistance phenomena with efficacy just under 30% in the patients [55]. The standard chemotherapy protocol, used just in patients with unresectable disease, is a combination of a platinum drug (cisplatin or carboplatin) and pemetrexed [132, 141], which is a coadjuvant to increase cytotoxicity.

The addition of antiangiogenic drugs such as bevacizumab [142] to this combination can increase the therapy's efficacy [136]. More recently, attention has shifted to immune checkpoint inhibitors (ICI), to activate the immune system to fight the disease. ICI uses monoclonal antibodies directed to specific receptors present on cytotoxic T‐cells [143], which inhibits their function against the cells of our body. By blocking these receptors (or their ligands) the T‐cells can exert their function when they recognise specific epitopes on the cancerous cells. In a randomised phase III study conducted on 605 patients, Baas *et al*. [144] showed that the administration of ipilimumab/nivolumab significantly increases the overall survival of the patients to 18 months, compared to 14 with chemotherapy alone. This is now a standard treatment for PMe in the USA, Australia, and the UK. The approval has nonetheless raised criticisms by several experts who have questioned the clinical and pharmaeconomic benefit of this approach compared to standard chemotherapy, following a thorough analysis [145, 146, 147, 148, 149]. There are also a few other experimental approaches that are currently being explored for the treatment of this pathology. For example, photodynamic treatment (PDT) is based on a chemical accumulated in tumour cells activated by a specific wavelength of light to produce reactive singlet oxygen (^1^O_2_). This technique showed potential as therapy after surgical resection [150].

Another option is the use of miRNA [150]: the overexpression of miR‐31 miR‐29c, or miR‐145^172^ in PMe cell lines has been shown to induce a decrease in proliferation, migration, invasion, and colony formation. Despite the promising findings *in vitro*, the only clinical study [151] so far completed failed to deliver conclusive positive results.

Finally, oncolytic viral therapy (OVT) involves the use of engineered viruses to kill the tumorigenic cells [152] via lysis and parallel stimulation of the immune system. In PMe, various viruses have been manipulated such as adenovirus, HSV Type 1, vaccinia virus, and measles virus. Even though clinical studies are still ongoing, OVT did not prove to be effective alone [153], but useful in combination with chemotherapy and surgery [154], thus indicating a possible adjuvant rather than curative for this approach.

The current lack of a standard of care for PMe highlights that a deeper knowledge of biology and its pathogenesis is indispensable to designing effective approaches, as well as designating standards for clinical trials to measure success.

## 7 Conclusion

The progress made in the comprehension of genetic and molecular mechanisms involved in the aetiopathogenesis of PMe has not yet evolved into therapeutic protocols or predictive biomarking strategies. This means that more needs to be done to explore the cell biology of the PMe's onset and progression. The right focus on securing translational impact has left several of the molecular hits, which recently emerged, not fully characterised. In the same way, those that have been used instead far more robustly linked with the disease are still in need of further clarification. This is epitomised by the evidence of the dual role acknowledged for BAP1, considered as a “friend” in healthy subjects, but as a “foe” in PMe patients. This is indicative that the full picture of the underlying dysregulated biology in PMe is not yet fully elucidated. However, the literature reviewed here highlights that in PMe physiopathology, two promising aspects of this disease remain understudied: signalling and metabolism. Despite the contributions on Ca^2+^ signalling, the role of other intracellular second messengers or respiration by‐products holding a similar role (e.g. ROS) remains unclear. Equally, a deeper knowledge of metabolic pathways and metabolites would better inform hidden oncological aspects of PMe, thus offering new avenues for personalised therapies. Formation and maintenance of a CSC population, tumour micromovements and T‐cells function are all strictly dependent on modified metabolism. In keeping with this, the dynamics of interorganelle contacts are also unexplored, which determine foci of metabolic and signalling events. Their full understanding will empower the ambition for tailored therapeutic strategies and improved diagnosis in this aggressive malignancy.

## Conflict of interest

The authors declare no conflicts of interest.

## Author contributions

Conceptualization, MR and MC; Writing: Original Draft, MR and MC; Writing: Review & Editing, MR, LM, and MC.
